# Beyond motivation: Creating supportive healthcare environments for engaging in therapeutic patient education according to healthcare providers

**DOI:** 10.1016/j.pecinn.2025.100405

**Published:** 2025-05-25

**Authors:** Bob C. Mulder, Hylkje Algra, Esther Cruijsen, J. Marianne Geleijnse, Renate M. Winkels, Willemieke Kroeze

**Affiliations:** a Strategic Communication group, Wageningen University and Research, Wageningen, the Netherlands; b Department of Nursing, Christian University of Applied Sciences (CHE), Ede, the Netherlands; c Division of Human Nutrition and Health, Wageningen University and Research, Wageningen, the Netherlands; d Nutrition & Healthcare Alliance, Ede, the Netherlands

**Keywords:** Therapeutic patient education, Lifestyle counselling, Health services research, Focus groups

## Abstract

**Objective:**

This article reports the findings of focus-group discussions with healthcare providers concerning the facilitators and barriers they experience when engaging in therapeutic patient education (TPE).

**Methods:**

Five focus-group discussions were held with a total of 21 primary and secondary healthcare providers. Discussions were moderated using a topic list that was co-created with healthcare providers. All discussions were recorded, transcribed verbatim and analysed thematically.

**Results:**

Healthcare providers consider TPE important, but it requires long-term, continuous effort in order to be effective. They sometimes doubt its effectiveness and their own efficacy. Moreover, healthcare providers experience a lack of a supportive environment. Overall, their experiences could be captured in four categories of determinants of engaging in TPE: Capabilities, Motivation, Physical Context and Social Context.

**Conclusion:**

Therapeutic patient education requires healthcare providers to be capable and motivated. To maintain the continuous effort needed, healthcare providers need to be supported both socially (e.g. by colleagues and management) and physically (e.g. through communication infrastructure).

**Innovation:**

In contrast to previous studies focusing on the motivation and capability of healthcare providers to perform TPE, this study contributes to innovation in health communication by identifying social and physical factors that determine whether TPE is delivered continuously under actual or perceived constraints in terms of time and effectiveness.

## Highlights

• Although healthcare professionals have a general positive attitude towards TPE in care, practice is lacking.
• Hesitance to discuss private matters and patient resistance hinder lifestyle counselling.
• A clear delineation of patient information about TPE is lacking between primary and secondary care.
• To improve professional practice, solutions must consider capability, motivation and social and physical contexts.

## 1 Introduction

Nutrition, smoking, physical activity and sleep are categories of lifestyle behaviours that are predictive of the development and progression of chronic diseases, including cardiovascular diseases [CVD] [1]. Lifestyle improvement is therefore an important component of guidelines for the prevention and treatment of CVD [2]. Despite the promising benefits of a healthy lifestyle, several studies have shown that patients with one or more chronic diseases continue to engage in unhealthy lifestyle choices after their initial diagnoses [3,4]. For example, patients with CVD often have an unhealthy lifestyle, as reflected in a high prevalence of smoking, sedentary behaviour and poor diet quality [5,6]. Reasons for the inability of patients to make lifestyle improvements include a lack of support, low motivation, low self-efficacy and a lack of knowledge or skills [7].

According to the WHO, *therapeutic patient education* (TPE) aims to support patients in managing chronic diseases, with a focus on healthy lifestyle [8]. It is defined as:

…education managed by health care providers trained in the education of patients, and designed to enable a patient (…) to manage the treatment of their condition and prevent avoidable complications, while maintaining or improving quality of life. Its principal purpose is to produce a therapeutic effect additional to that of all other interventions (pharmacological, physical therapy, etc.) ([8], p. 5).

In line with the adjective ‘therapeutic’, TPE has been found to improve health in patients with chronic diseases [9,10]. Patients value TPE when it involves open dialogue – meaning honest and non-judgmental, straight to the point, caring and in easily comprehensible language. Such communication helps patients to feel satisfied with their care, more confident and less worried, knowledgeable and supported, in addition to making them more receptive to adopting healthier behaviours [11].

Educating patients about healthy lifestyle is still far from common practice, and not only because of a lack of time or resources. Studies on why healthcare providers do (or do not) engage in TPE reveal a number of common determinants. [[12], [13], [14], [15], [16], [17], [18], [19], [20]]. One issue has to do with a decline in motivation that can occur when a healthcare provider does not immediately see a change in the patient's behaviour in response to TPE [14]. Doubts concerning the effectiveness of health promotion can thus be a barrier that impedes healthcare providers from practicing TPE [20]. In addition, healthcare providers have reported lacking the required knowledge and skills; having suboptimal lifestyles themselves; or fearing that TPE might strain the relationship with the patient [16,17]. In contrast, factors that facilitate the efforts of healthcare providers to engage in TPE include current theoretical and clinical knowledge; advanced communication skills, (including the ability to establish interpersonal relationships with patients); meeting their learning needs; facilitating effective dialogue; and providing individualised patient-centred education and lifestyle counselling [13,15,18]. Healthcare providers also view TPE positively when it meets their need to shift the focus of their work from technical-medical care towards building relationships with patients and supporting them in self-management [14]. When healthcare providers perceive TPE as important, effective and as part of their role, this increases the likelihood that they will engage in TPE [19].

In addition to individual factors, other barriers to engaging in TPE relate to collaborations between healthcare providers. Due to the complexity of chronic diseases, several providers are involved in the treatment of a patient. For example, the treatment of patients with CVD involves providers from both primary care (e.g. general practitioners, nurse practitioners) and secondary (i.e. hospital) care (e.g. nurses, internists, cardiologists). Given that the promotion of a healthy lifestyle is part of the treatment guidelines for all of these healthcare providers [2,21], communication between providers is essential to helping patients self-manage their lifestyles [22]. During the preparation of the present study, healthcare professionals indicated the relevance of professional collaboration within and across the boundaries of primary and secondary care when promoting a healthy lifestyle to their patients, as well as the need to explore how to improve this collaboration [program manager Chronic Care from a regional GP organisation, personal communication, March 2020]. They nevertheless experience a lack of sufficient financial means and external options for referring patients to other medical or paramedical organisations [20].

To address the sparsity of literature on this topic, we further explore personal and environmental facilitators of and barriers to initiating TPE during regular consultations. The idea for this research arose in consultation with healthcare providers who were attempting to integrate TPE into their daily practice. This study pertains specifically to the Dutch context, within which preventive healthcare has been gaining both political and public attention. We expect that the attitudes and actual performance of healthcare providers are likely to vary according to personal factors, as well as to actual or perceived social and physical factors. The objective of this research is therefore to explore the attitudes and motivations of healthcare providers concerning TPE, as well as their experiences with practicing TPE. We also explore the views of healthcare providers on collaboration between primary and secondary care within the context of TPE.

## 2 Methods

### 2.1 Design

This study is based on focus groups with healthcare providers. This methodology was selected because focus groups are particularly well-suited to exploring how attitudes and experiences are constructed and shaped within a shared cultural context – in this case, the healthcare system. Given that the participants were all working within the same context, they were able to encourage each other to explore their views by exchanging anecdotes, responding to each other and asking questions. In this way, the participants constructed a shared understanding of what lifestyle counselling means in a healthcare setting [23,24].

In addition to the focus groups, a document analysis was conducted to generate insight into the content and wording of the various guidelines and agreements relating to TPE in both primary and secondary care. We created an overview of the tasks and responsibilities of various healthcare providers (e.g. doctors and nurses) in relation to TPE, as well as the ways in which these professionals should perform TPE. We further identified ambiguities in and differences between guidelines.

### 2.2 Participants

Healthcare providers working in primary care were invited to participate through the newsletters of a GP organisation in a central region of the Netherlands. Healthcare providers working in secondary care were recruited from a large peripheral hospital by a researcher working there. For both settings, recruitment focused on including both nurses and medical doctors. Hospital-based healthcare providers were eligible if they worked with patients treated for cardiovascular risk management (CVRM) – for example, patients with CVD or diabetes. The invitation was thus extended to all healthcare providers from the fields of internal medicine, cardiovascular medicine and neurology. Those expressing interest in participating were provided with a description of the study and contact details for the research team.

In all, 21 healthcare providers from both primary and secondary care participated in five focus-group sessions. Three focus groups were held with a total of four general practitioners (GPs) and nine practice nurses working in primary care. Two focus groups were conducted with six specialists (two cardiologists, three internists and one neurologist) and two nurses (one diabetes nurse specialist and one cardiac nurse specialist) from the hospital.

### 2.3 Data collection

The focus groups were conducted between September 2020 and June 2021. Because of COVID-19 restrictions and other practicalities, the sessions were conducted online, with the exception of the first focus group with primary healthcare providers. Before the focus-group session, participants completed a short questionnaire, which included items on their age, gender, education – both general and specifically focused on lifestyle and health – and several aspects of their jobs. All sessions were recorded using the recording function of MS Teams.

The focus groups were moderated by two trained facilitators (authors WK and BCM), according to a topic list based on theory and evidence, with a focus on three main themes: attitudes towards TPE (e.g. expected benefits, enjoyment when performing TPE), practicing TPE (e.g. how to decide on initiating TPE, how to motivate patients to make lifestyle changes) and collaboration within and between primary and secondary care within the context of TPE (e.g. views on roles and task distributions within TPE or on how to incorporate TPE into referrals). The topic list was pre-tested by a member of a network organisation for GP care, to ensure that the questions were clear, relevant to practice and worded with the right tone of voice. The focus groups with primary healthcare providers were held first, followed by those with hospital-based providers. Each session lasted an average of 60 min.

### 2.4 Data analysis

Recordings of the focus groups were transcribed verbatim by a transcription service and subsequently coded using Atlas.ti (version 22). During the transcription process, privacy-sensitive details (e.g. names) were omitted. Authors WK and BM independently coded all transcripts. A set of deductive codes was initially derived from the topic list, and it served as the starting point for both coders. Additional inductive codes were introduced throughout the coding process. Following the approach proposed by Kiger and Varpio [25], coding was viewed as a crucial intermediary step, aimed at identifying and emphasising key elements within the data, which formed the basis for identifying overarching themes. Each coder developed a thematic framework, which the two coders then compared and discussed. This resulted in minor refinement of the thematic framework and consensus on its content.

### 2.5 Discussion meeting for solutions

After the framework was developed, a voluntary selection of the focus-group participants (3 from primary care, 3 from secondary care) participated in a discussion meeting. The focus of this meeting was on possible solutions for the issues emerging from the focus groups and document analyses. In preparation for this meeting, participants received a factsheet containing a summary of the results. During the meeting, the discussion focused on the following themes: the need for educational material, communication between primary and secondary healthcare providers, the tasks and responsibilities of healthcare providers, and unambiguous advice by healthcare providers. The outcomes of this session were used to derive the practical implications presented in the Discussion section of this article.

### 2.6 Ethics

The study protocol was reviewed by the Social Sciences Ethics Committee [blinded for review], who concluded that the protocol deals with ethical issues in a satisfactory manner and complies with the Netherlands Code of Conduct for Research Integrity. All participants were informed about the study in writing, and all signed statements of informed consent before participating in the study.

## 3 Results

During the focus groups, participants freely discussed their views and experiences on TPE, both positive and negative, within an open, collegial atmosphere characterised by humour and critical self-reflection. The participants perceived the joint reflection as supportive, given the high motivational potential of sharing success stories. They also had the sense that they were not alone in sometimes experiencing struggles with TPE, including having doubts concerning its effectiveness.

**Fig. 1:**
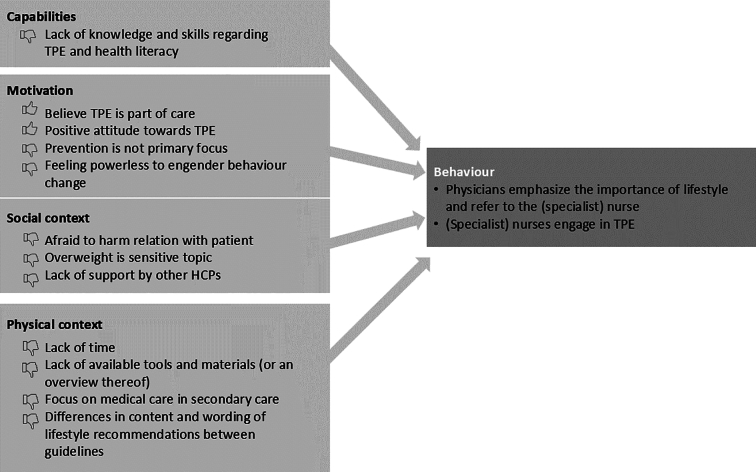
Barriers and facilitators for engaging in therapeutic patient education.

One prominent theme expressed by the healthcare providers in the focus groups is that TPE requires long-term, continuous effort in order to be effective. Our analyses further revealed that engaging in TPE is determined by four categories of determinants (see Fig. 1 for an overview): Capabilities and Motivation at the individual level; and Social Context and Physical Context at the environmental level.

The categories of Capabilities and Motivation contain factors that can either hinder or facilitate engagement in TPE. In terms of capabilities, the participants reported experiencing a lack of knowledge and skills needed in order to be effective in educating patients about lifestyle:

(…) but that's like knocking on open doors: exercising for half an hour five times a week, perhaps participating in a sport if possible. Start small as [name of participant] says, give only really good advice or real scientific knowledge or something to back it up. I'm not really sure, so – again – I don't think I have that knowledge. [Focus Group 4, secondary care].

In addition to a perceived lack of lifestyle knowledge, participants reported experiencing challenges in finding effective ways of communicating with patients in a manner that encourages them to be open to talking about lifestyle and changing their lifestyles accordingly:

So that's exactly what makes it incredibly difficult and, actually, over time, I've increasingly started looking, not for ‘watch out, because in 10 years you'll have a stroke or a heart attack’, but for something like ‘if you do it now, you'll feel better, feel fitter’. A bit of the positive, that's what I'm really looking for to motivate people, but it's just very difficult. [Focus Group 2, primary care].

The participants found TPE with patients with low health literacy to be particularly challenging:

We also have to deal with people with low literacy. We also notice that… I sometimes think, ‘I don't think people understand it. Do they know what they're reading? Can they read it?’ (Focus Group 4, secondary care).

The motivation of healthcare providers was positively related to the subthemes of ‘Believing TPE is part of care’ and ‘Having a positive attitude’. It was negatively related to ‘Prevention is not the primary focus’ and ‘Feeling powerless to engender behaviour change’. While some participants strongly believed that TPE is part of healthcare, others expressed doubts:

That's our core business: lifestyle. [Focus Group 2, primary care].

There's always the question of whether it's our job. Providing health education and training, and encouraging exercise could also be a societal function. I think that the municipality could play a very big role in this regard. We don't have to want to solve everything either. [Focus Group 1, primary care].

Despite these differences, most participants expressed positive attitudes towards TPE, due to its impact on disease burden and quality of life for patients.

In that way, I think that we – as doctors – play a very important role, both as role models and as advisors for the people in our consultation rooms. Even if we meet them at the end of a disease trajectory, because [even if] the effects of preventive measures are no longer so great, I still think that, as doctors, we play a very important role in that, including in hospital. [Focus Group 5, secondary care].

(…), but our wish has always been to address that lifestyle. [Focus Group 3, primary care].

Some participants also expressed the opinion that prevention is not their primary focus, as clinical and/or medical care often take precedence.

We are ‘request-for-help’ doctors. The patient comes in, and the assistant asks, ‘Why are you coming in to see the doctor?’ We thus give time to that, and how much time do we have to deal with that? There has to be something related to lifestyle, or if a question happens to arise, then it comes up. [Focus Group 1, primary care].

Although prevention is not the main part of our work. We're concerned with treating problems that can be solved. It's very much treatment-oriented, and much less prevention-oriented than in primary care, but prevention is obviously part of your work. [Focus Group 4, secondary care].

Finally, some healthcare providers experience powerlessness, as the patient is the one making the choice for behaviour change. They often see that patients fail to make lifestyle changes:

You can only invite patients to be willing to change. You can't change them, so this ultimately remains the patients' choice. And we keep inviting them; we do that, and we inform them and that's it. [Focus Group 1, primary care].

The social context and the physical context influence the behaviour of healthcare providers, even beyond their own individual capabilities and motivation. Socially, healthcare providers must combine providing lifestyle advice with managing their relationships with patients, which they are reluctant to harm. Healthcare providers may encounter resistance from patients, but they may also feel resistance themselves with regard to entering the private lives of patients, which is inherent to discussions of lifestyle choices.

I can't tell everyone to stop smoking. They find that very annoying. (…) Not everyone likes that when there's no connection with the complaint. [Moderator]: How do you feel as a healthcare professional if you have started that conversation and you experience such resistance? [HCP]: I don't like that. I think that you get resistance because of that, and no cooperation, but rather distance. It also raises a threshold for the next time. Unsolicited advice is not always appreciated. [Focus Group 1, primary care].

This barrier is exacerbated by the fact that overweight is seen as a sensitive topic:

Being heavy is so ingrained in people. They build that up for years, and they're also fat for years. That doesn't happen from one day to the next. It thus becomes a part of their identity. People take it much more as a personal remark. I always find that remarkable. That's why I sometimes have trouble starting to talk about it. [Focus Group 5, secondary care].

Some healthcare providers also perceive that their colleagues not provide sufficient support for TPE. Healthcare providers sometimes don't know what their colleagues do regarding TPE due to lack of communication between primary and secondary care. Besides, lack of support might be due to the wide variation amongst healthcare providers in terms of attitudes about and approaches to TPE:

I had to laugh a lot about what came out – completely different thoughts, completely different mentalities about it. That one patient who goes to those three healthcare providers isn't going to lose weight, isn't going to stop smoking, because the patient will remain frustrated. Then I thought, we've got to tackle our own mentality first, so that everyone says the same thing. [Focus Group 1, primary care].

In addition to social factors, healthcare providers may perceive the influence of the Physical context on their performance of TPE. This context could be captured under the subthemes ‘Lack of time’, ‘Lack of available tools and materials (or an overview thereof)’, and ‘Focus on medical care in secondary care’. In addition, the document analysis revealed differences in the content and wording of lifestyle recommendations in the various guidelines.

Lack of time was often mentioned and, perhaps not surprisingly, related to financial issues:

Everything has to do with time. If I'm running late, I do less of that and more with regard to the patient's complaint –especially with regard to what the patient wants. If they want to talk about it, I'll have time for that, but that's usually not the initial complaint, so it's always something on the sidelines. (Focus Group 1, primary care).

That 5-and-10-min mentality that's constantly being forced upon us, it's difficult. [Moderator]: By that you mean the consultation time. [HCP]: That really makes me despondent, and the practice nurse as well. The practice nurse and I are going to see the patients. (…), you demand that kind of quality, but at the end of the month, you demand that the practice nurse has seen all of the CVRM patients, because they must have all had an annual check-up and a three-month check-up. Otherwise, you get less money. The practice nurse is thus also under pressure. I think this is more of a system problem. [Focus Group 1, primary care].

Participants reported experiencing a lack of available tools and materials (and/or a lack of an overview of such materials) that can meet the diverse needs and preferences of their patients:

We are still highly focused on leaflets, but I think you can do much more with images and videos to clarify it. We have a target group; they come in at 18 [years of age], but we also have them from 90 [years of age], and there are huge differences in level and motivation. You really have to approach it in different ways if you want to reach all those people well. [Focus Group 4, secondary care].

Another factor is that primary healthcare providers perceive that their colleagues in secondary care do not pay much attention to lifestyle. This observation was surprising, and it pointed to a lack of continuity throughout the process of educating patients about lifestyle:

I've noticed that the secondary line has little compassion for lifestyle. I sometimes ask people, ‘Has the cardiologist talked to you about your lifestyle, about smoking – because you smoke – or about nutrition?’ ‘No, he never talks about that’. I hear this not only once, but very often. [Focus Group 2, primary care].

I think that, when internists refer their patients back to the GP and we, as practice assistants, start working with them, I sometimes have the idea that I just have a new patient – that I have to start from the beginning. Then I wonder, ‘What's happening there?’ But it obviously might not be entirely fair to say it that way. [Focus Group 3, primary care].

Finally, as revealed by the document analysis, differences in the content and wording of lifestyle recommendations in the various guidelines can lead to confusion. For example, according to national CVRM guidelines, patients should be advised to consume more fruit and vegetables, and to restrict their intake of salt, saturated fat and cholesterol. In contrast, Regional Transmural Agreements, which document communication and referrals between primary and secondary care within a specific region, do not mention saturated fat or cholesterol, but instead recommend that patients with hypertension should eat a healthy diet, restrict their salt intake and not consume liquorice. Such differences hinder clear communication within and between lines of care, and they can lead patients to perceive that healthcare providers are giving different advice with regard to lifestyle. This may cause confusion, thereby having a negative effect on patient motivation.

## 4 Discussion and conclusion

### 4.1 Discussion

The objective of this study was to explore the views and experiences of healthcare providers with regard to TPE, focusing specifically on their individual attitudes and motivations, as well as on social and physical factors that may either hinder or facilitate their engagement in TPE. The findings were synthesised into four main themes: Capabilities and Motivation (at the individual level) and Social Context and Physical Context (at the environmental level).

The themes of Capabilities and Motivation correspond to previous studies focusing on determinants of the motivation of healthcare providers to engage in TPE [[12], [13], [14], [15], [16], [17], [18], [19], [20]]. The theme of Capabilities was largely dominated by the subtheme ‘Lack of knowledge and skills regarding the actual recommended lifestyle’ (e.g. dietary guidelines). The healthcare providers participating in our study also reported experiencing difficulty communicating with patients in a way that motivates them to change their behaviour. The education of patients with low levels of health literacy was perceived as particularly challenging. This factor was related to the motivation of healthcare providers to engage in TPE, as feeling powerless to guide patients towards actual behaviour change can have a negative effect on their motivation. Another negative factor was the curative focus of their consultations with patients, which contrasted with their belief that TPE is an integral part of healthcare, as well as with their generally positive attitude towards TPE. This finding suggests that most healthcare providers are likely to experience some level of ambivalence towards TPE.

Although motivation is obviously necessary, the results of this study indicate that motivation alone is not sufficient to ensure that healthcare providers will be able to perform TPE continuously. They need social and physical contexts that support and facilitate lifestyle counselling. These factors that have not received much attention in previous studies. Most factors relating to the social context are negative, relating to perceptions that colleagues have varying attitudes and approaches to TPE, as well as to the perception that TPE may harm their relationships with patients (especially those with overweight). Factors identified in relation to the physical context were largely hindering as well, including lack of time, lack of tools and materials (or an overview thereof) for educating patients, and a focus on medical care in the secondary line that hinders collaboration and coordination between lines of care.

Based on these findings, and in collaboration with a voluntary selection of the professionals who had participated in the focus groups, we formulated recommendations for creating a healthcare environment that is more supportive of TPE. These recommendations were made at the level of healthcare providers and, notably, at the level of healthcare organisations that have the power and responsibility to create environments that are supportive of TPE.

First, healthcare providers are advised to persevere in addressing lifestyle, adopting a planned approach, and ideally using visual materials, especially for patients with low levels of health literacy. This recommendation confirms that TPE is an integral component of care. It focuses on the process of performing TPE, as opposed to focusing on patient-level outcomes (which can be frustrating). One notable example of such patient-level outcomes has to do with behaviour change and/or clinical improvement (or the lack thereof).

Second, healthcare providers require supportive contexts within which to initiate and maintain TPE. They stand to benefit from the support of their colleagues and the acknowledgement of the relevance of addressing lifestyle. More specifically, motivation and collaboration could be improved by regularly meeting other healthcare providers to share experiences and success stories. At the organizational level, care organisations should improve collaboration (e.g. through transmural agreements) in which TPE responsibilities are described and communication about TPE is facilitated. This requires referral systems that contain entry fields to describe what has been done in terms of TPE. In addition, uniform TPE materials and tools are needed, with comprehensive and uniform lifestyle guidelines that are clear to both patients and healthcare providers.

Finally, at the national level, challenges regarding financing should be addressed with insurance companies, and TPE skills should be included as a standard part of curricula for medical education and training.

### 4.2 Innovation

Even though the importance of TPE to the self-management of lifestyle-related chronic diseases is undebated, the practice of TPE is generally not embedded into standard healthcare protocols. As such, TPE requires continuous effort from healthcare providers, and its effectiveness thus depends on their motivation, as reported in previous studies [[12], [13], [14], [15], [16], [17], [18], [19], [20]]. Our findings confirm the importance of motivation and its determinants, but they also highlight factors extending beyond the individual level. Social and physical factors in the work environment are equally important to support healthcare providers in their efforts to engage in TPE, as also observed in a recently published study [26]. These results suggest that the decisions of healthcare providers to introduce (or re-introduce) the topic of lifestyle with their patients are heavily influenced by their perceptions concerning whether TPE is or is not an integral part of ‘standard’ care (and thus whether it is or is not also practiced by their colleagues). Another indication of TPE as a component of standard care is whether electronic patient records do or do not contain entry fields for noting what has been discussed with patients in terms of lifestyle.

### 4.3 Conclusion

Although many healthcare providers are motivated to discuss lifestyle with their patients, TPE demands more from these professionals than motivation alone. The application of TPE in practice requires changes and collaborations at multiple levels of the healthcare system, and even in society. More specifically, TPE cannot be effective unless healthcare providers are sufficiently trained, and unless they are supported by their colleagues and the wider organisations within which they work. If TPE is accepted as normal and even necessary, it should be embedded within the prevailing systems of remuneration and communication. Such system changes could translate into societal change, such that patients will come to expect their healthcare providers to bring up the topic of lifestyle during consultations.

## CRediT authorship contribution statement

**Bob C. Mulder:** Methodology, Formal analysis, Conceptualization, Writing – review & editing, Writing – original draft. **Hylkje Algra:** Visualization, Project administration, Investigation, Formal analysis, Writing – review & editing, Writing – original draft. **J. Marianne Geleijnse:** Supervision, Resources, Funding acquisition, Writing – review & editing. **Renate M. Winkels:** Funding acquisition, Formal analysis, Writing – review & editing, Writing – original draft. **Willemieke Kroeze:** Resources, Project administration, Funding acquisition, Formal analysis, Writing – review & editing, Writing – original draft. **Esther Cruijsen:** Writing – review & editing.

## Declaration of competing interest

The authors declare that they have no known competing financial interests or personal relationships that could have appeared to influence the work reported in this paper.

The authors declare the following financial interests/personal relationships which may be considered as potential competing interests:

Bob C Mulder reports financial support was provided by Wageningen University. If there are other authors, they declare that they have no known competing financial interests or personal relationships that could have appeared to influence the work reported in this paper
